# Supplementary material for: DMRforPairs: identifying Differentially Methylated Regions between unique samples using array based methylation profiles
Source: BMC Bioinformatics. 2014 May 15;15:141. doi: 10.1186/1471-2105-15-141 (PMC4046028; doi:10.1186/1471-2105-15-141)
Supplement: Additional file 2 — DMRforPairs output for the comparison of A431-MCF7 and NA17018-NA17105. Please start from the HTML files in each folder. Available via the BMC Bioinformatics website. [file 1471-2105-15-141-S2.zip › 1394847754114233_MOESM2_ESM/A431_MCF7/figures/39474.pdf]

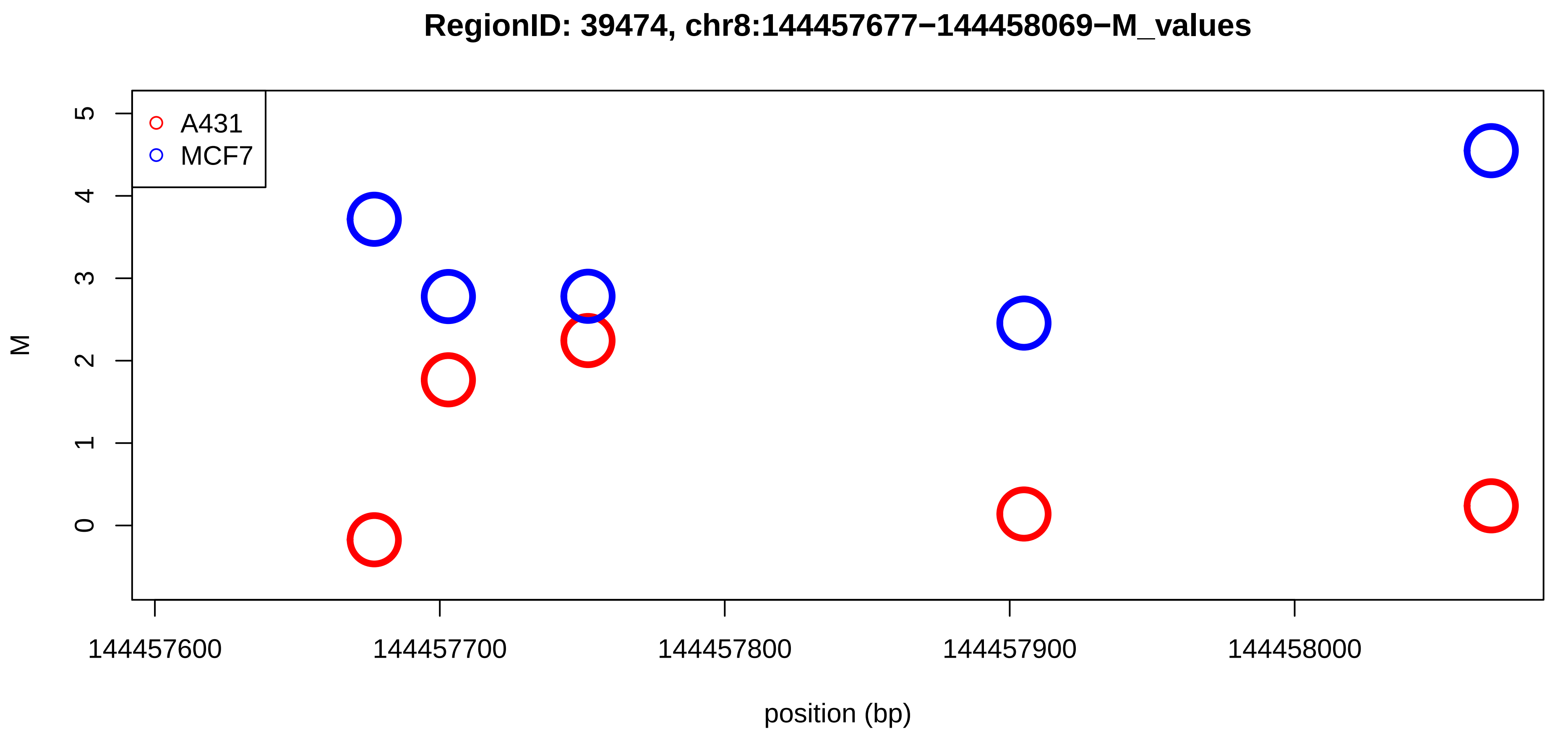

RegionID: 39474, chr8:144457677−144458069−M_values
A431
MCF7
M
0
1
2
3
4
5
144457600
144457700
144457800
144457900
144458000
position (bp)


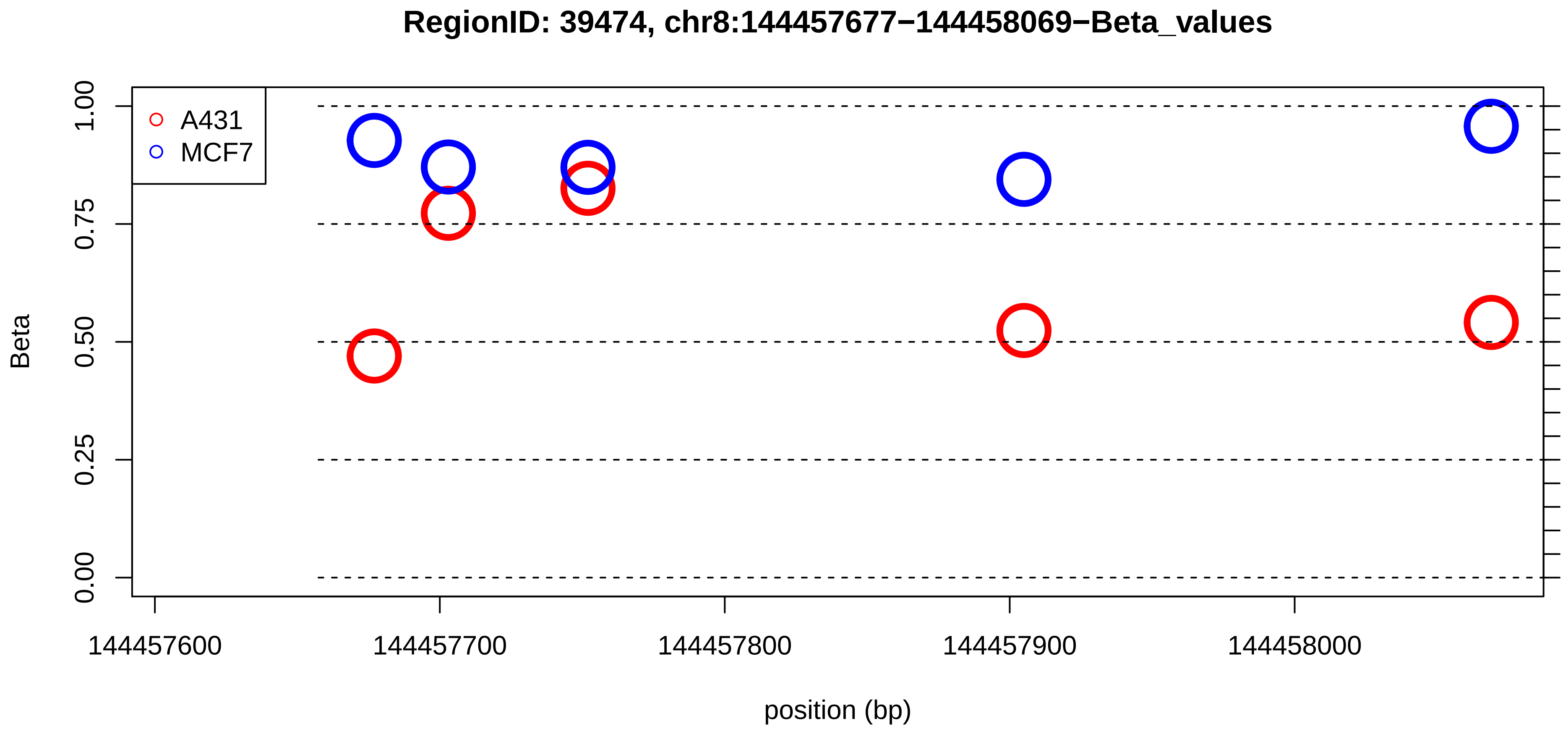

RegionID: 39474, chr8:144457677−144458069−Beta_values
A431
MCF7
Beta
0.00
0.25
0.50
0.75
1.00
144457600
144457700
144457800
144457900
144458000
position (bp)

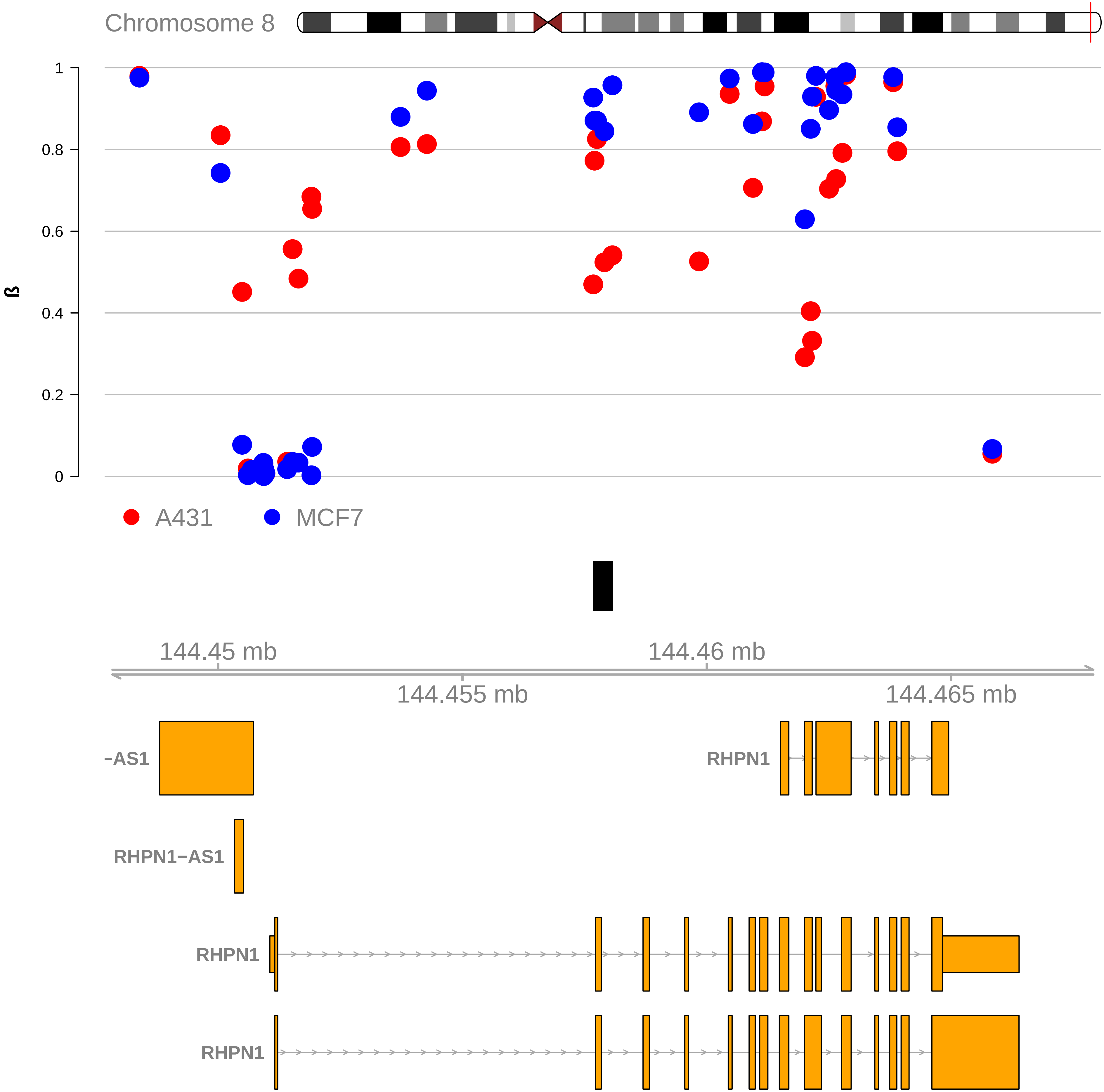

Chromosome 8
1
0.8
0.6
0.4
0.2
0
ß
A431
MCF7
144.45 mb
144.46 mb
144.455 mb
144.465 mb
–AS1
RHPN1
RHPN1–AS1
RHPN1
RHPN1
